# SGLT2i reduce arrhythmic events in heart failure patients with cardiac implantable electronic devices

**DOI:** 10.1002/ehf2.15223

**Published:** 2025-02-07

**Authors:** Marco Valerio Mariani, Carlo Lavalle, Marta Palombi, Nicola Pierucci, Sara Trivigno, Andrea D'Amato, Domenico Filomena, Pietro Cipollone, Domenico Laviola, Agostino Piro, Silvia Prosperi, Josefina Magliolo, Vincenzo Myftari, Vincenzo Mirco La Fazia, Paolo Severino, Cristina Chimenti, Roberto Badagliacca, Carmine Dario Vizza

**Affiliations:** ^1^ Department of Cardiovascular, Respiratory, Nephrological, Aenesthesiological and Geriatric Sciences ‘Sapienza’ University of Rome Rome Italy; ^2^ St. David's Medical Center Texas Cardiac Arrhythmia Institute Austin Texas USA

**Keywords:** arrhythmias, cardiac implantable electronic devices, heart failure, remote monitoring, sodium glucose cotransporter 2 inhibitors

## Abstract

**Background:**

Sodium glucose cotransporter 2 inhibitors (SGLT2i) represent one of the four pillars of heart failure (HF) pharmacological therapy.

**Objective:**

The study aims to clarify SGLT2i antiarrhythmic effect on patients with HF with reduced ejection fraction (HFrEF) in terms of atrial and ventricular arrhythmias (AAs and VAs) reduction.

**Methods:**

HFrEF carriers of implantable cardioverter defibrillator (ICD) or cardiac resynchronization therapy defibrillator (CRT‐D) followed by remote monitoring of Policlinico Umberto I of Rome for 1 year before and after SGLT2i therapy initiation were enrolled in the study. We compared the incidence of AAs and VAs as recorded at remote monitoring during 1 year preceding SGLT2i therapy initiation and after 1 year of SGLT2i therapy.

**Results:**

Among 198 enrolled patients, 135 patients had arrhythmic events before SGLT2i therapy prescription. There were 1353 arrhythmic events recorded in the year before SGLT2i therapy prescription, and 354 events were detected in the year after SGLT2i initiation, with a 73.8% reduction in events number after therapy initiation. After SGLT2i therapy initiation, the median number of total arrhythmic episodes significantly decreased from a median of 7 [3;12] to 1 [0;4] (*P* value < 0.001), AAs significantly decreased from a median of 4 [3;7] to 1 [0;3] episodes (*P* value < 0.001) and VAs were reduced from a median of 5.5 [3;10] to 0 [0;2] (*P* value < 0.001). When considering arrhythmia subtypes, larger reductions were recorded for atrial fibrillation (AF) episodes, reduced from 4 [3;8] to 0 [0;3], non‐sustained ventricular tachycardia (NSVT) that decreased from 4 [2;8.75] to 0 [0;2] (*P* value < 0.001) and for sustained ventricular tachycardia (SVT) that were reduced from 3 [2;4] to 0 [0;1] (*P* value < 0.001).

**Conclusions:**

In HFrEF carriers of ICD/CRT‐D, the use of SGLT2i resulted in significant reduction of AA and VA events.

## Introduction

Heart failure (HF) syndrome is associated with the development of atrial and ventricular arrhythmias.^1^, ^2^ When HF patients develop atrial fibrillation (AF), the risk of adverse cardiovascular (CV) outcomes, such as CV mortality and HF‐related hospitalization (HFH), significantly increases.^3^ Ventricular arrhythmias (VAs) are common in HF patients and sustained VAs are the leading cause of sudden cardiac death (SCD) in this population.^4^ Considering the consequences of atrial arrhythmias (AAs) and VAs in HF patients, upstream therapy capable of preventing arrhythmia occurrence and/or reducing arrhythmic burden is pivotal and should be pursued. Alongside beta‐blockers, HF drugs such as angiotensin‐converting enzyme inhibitors (ACEi)/angiotensin receptor blockers (ARBs), mineralocorticoid receptor antagonists (MRAs) are non‐antiarrhythmic drugs with antiarrhythmic properties and showed to reduce both AAs and VAs.^5^ On the same line, angiotensin receptor‐neprilysin inhibitor (ARNI) demonstrated to significantly reduce the risk of AF progression as compared with ARB in a propensity‐matched cohort and to reduce VAs and the risk of SCD in a post‐hoc analysis of the PARADIGM‐HF trial.^6^, ^7^ Sodium glucose cotransporter 2 inhibitors (SGLT2i) represent one of the four pillars of HF pharmacological therapy and are recommended by current guidelines in all patients with HF, regardless the LVEF.^8^ In a meta‐analysis of randomized controlled trials (RCTs), SGLT2i have demonstrated to reduce AF occurrence, whereas different studies have been inconclusive in showing a significant reduction in SCD and VAs with SGLT2i therapy.^9^, ^10^ Noteworthy, arrhythmic events were reported as serious adverse events and not as primary endpoint event in all currently available RCTs on SGLT2i, possibly leading to underreporting bias. Therefore, the anti‐arrhythmic effect of SGLT2i has not been clearly demonstrated yet.

With the aim to clarify SGLT2i antiarrhythmic effect, we performed a real‐world study evaluating AAs and VAs in HF patients with reduced ejection fraction (HFrEF) and an ICD/CRT‐D followed by remote monitoring.

## Material and methods

**Figure 1:**
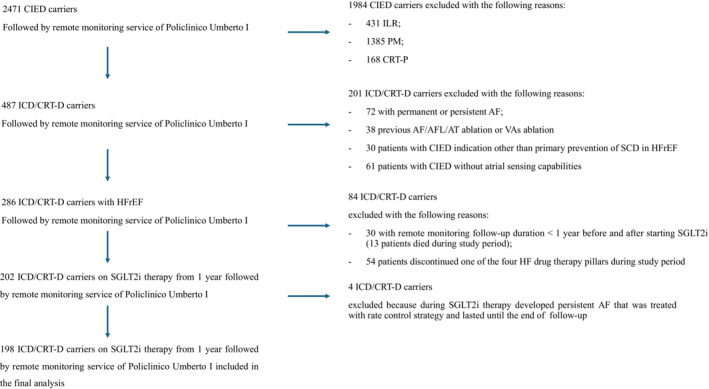
Diagram of patients' selection. AF, atrial fibrillation; AFL, atrial flutter; AT, atrial tachycardia; CIED, cardiac implantable electronic device; CRT‐D, cardiac resynchronization therapy – defibrillator; CRT‐P, cardiac resynchronization therapy‐pacemaker; HF, heart failure; HFrEF, heart failure with reduced ejection fraction; ICD, implantable cardioverter defibrillator; ILR, implantable loop recorder; PM, pacemaker; SCD, sudden cardiac death; SGLT2i, sodium glucose cotransporter 2 inhibitor; VA, ventricular arrhythmia.

Patient selection and study flowchart are reported in *Figure* 1 and graphical abstract, respectively. All cardiac implantable electronic device (CIED) carriers followed by remote monitoring service in Policlinico Umberto I Hospital of Rome were retrospectively screened. Subsequently, the following exclusion criteria were applied: patients with implantable loop recorder or pacemaker or cardiac resynchronization therapy‐pacemaker; patients with permanent of persistent AF, carriers of CIED without atrial sensing capabilities, previous AF or VA ablation procedure; carriers of ICD/CRT‐D implanted in secondary prevention or for heart disease other than HFrEF. Moreover, patients with a remote monitoring follow‐up duration <1 year before and after the initiation of SGLT2i were excluded, as well as patients who discontinued one of the four HF drug therapy pillars after the SGLT2i therapy initiation. Eventually, HFrEF carriers of ICD/CRT‐D implanted for primary prevention of SCD, in stable OMT, and followed by remote monitoring of Policlinico Umberto I of Rome for 1 year before and after SGLT2i therapy initiation was enrolled in the study.

For each patient, demographic, clinical, laboratory and echocardiographic data were collected, as well as remote monitoring interrogation reports and SGLT2i therapy initiation date. In particular, the summary report with all device interrogation reports, including arrhythmic episodes, were reviewed and downloaded from the dedicated remote monitoring platform of each CIED brand by board certified electrophysiologists. All episodes recorded during the year before and after SGLT2i prescription were considered. Subsequently, the episodes were divided in arrhythmic episodes and non‐arrhythmic episodes. The latter were considered associated with lead or generator malfunction, lead fracture, over‐sensing, electromagnetic interference or sinus tachycardia. Arrhythmic episodes were divided in AAs and VAs. AAs were defined as any CIED detected episode lasting ≥30 s and were classified as AF, atrial flutter (AFL), atrial tachycardias (ATs), atrial high rate events (AHREs) or subclinical AF (in patients without a previous diagnosis of AF). For ease of the study, AF episodes and AHRE/subclinical AF episodes were collected as a unique category of AAs. VAs were divided in non‐sustained ventricular tachycardias (NSVTs), sustained ventricular tachycardias (SVTs), ventricular fibrillation (VF) and VAs requiring ICD therapy [anti‐tachycardia pacing (ATP) or shock]. Because our population included primary prevention ICD carriers, we programmed long detection window according to the recommendations of the expert consensus statement on optimal ICD programming and testing.^11^ All CIED were programmed with a non‐therapy zone (VT1) for tachycardia monitoring; a VT detection zone (VT2) and a VF detection zone were programmed according to manufacturer‐specific translation of ICD programming recommendations.^12^ NSVT was defined as a device‐detected ventricular event with a heart rate higher the lowest programmed VT detection rate (VT1) and an estimated duration of less than 30 s; SVT was defined as a device‐detected ventricular event in VT detection zones and an estimated duration of more than 30 s; VF was defined as a device‐detected ventricular event in VF detection zone; VAs requiring ICD therapy were defined as ventricular events treated with ATP/shock delivery.

We compared the incidence of AAs and VAs as recorded at remote monitoring during 1 year preceding SGLT2i therapy initiation and after 1 year of SGLT2i therapy. The primary outcomes were (1) the median number of total arrhythmic events recorded during the year before and after SGLT2i therapy initiation in patients with baseline arrhythmias off SGLT2i therapy; (2) the median number of each subtype of AA and VA recorded during the year before and after SGLT2i therapy initiation in patients with arrhythmic events detected at remote monitoring off SGLT2i therapy. The secondary outcomes were (1) the median number of arrhythmic events recorded in the overall population during the year before and after SGLT2i therapy initiation; (2) the median number of each subtype of AA and VA in total population; (3) the median number of each subtype of AA and VA in subgroups of interest based on SGLT2i indication and device type: patients with or without diabetes mellitus type 2 (T2DM), ischaemic or non‐ischaemic cardiomyopathy and device type (ICD or CRT‐D). The study was approved by the local ethical committee.

## Statistical analysis

The normal distribution of variables was assessed with the Shapiro–Wilk test. Continuous variables were expressed as mean and standard deviation, whereas median, first and third quartile were used for non‐normally distributed data. Categorical data were described as the number and percentage. Student's *t*‐test, Mann–Whitney test, the *χ* ^2^ test and the Fisher exact test were used for comparisons, as needed. Bar graphs and box plots were used to visually report study results. For all tests, a *P* value < 0.05 was considered statistically significant.

The statistical analysis was performed using SPSS version 27.0 for Mac (IBM Software, Inc., Armonk, NY, USA).

## Results

### Study population

Among 2471 CIED carriers followed by remote monitoring service of Policlinico Umberto I Hospital of Rome, 198 HFrEF patients implanted with an ICD or CRT‐D, on stable OMT and taking one of the available SGLT2i for at least 1 year were included in the study. Patients' selection and study population features are reported in *Figure* 1 and *Table* 1, respectively. Median age was 72.5 years (65;78), with 171 (86.4%) male patients. The prevalence of T2DM was 38% (*n* = 75) and chronic kidney disease (CKD) was present in 70% of patients. Ischaemic cardiomyopathy was the most common cause of HFrEF (62.2%) and median LVEF was 30% (22.5;35). AF/AFL history was present in 44% (*n* = 87). Dapagliflozin was prescribed in 69.7% of patients (*n* = 138), whereas the remaining 30.3% of patients (*n* = 60) were prescribed with empagliflozin. OMT was prescribed at the maximum tolerated dose in all patients, unless contraindicated or not tolerated. Of note, as per‐protocol definition, patients who discontinued one of the four pillars of HF drug therapy were excluded from the analysis; thus, no difference in terms of OMT prescription was present before and after SGLT2i prescription (*Table* 2). Antiarrhythmic drugs (AADs) prescription rate was low in the year before SGLT2i prescription, 12 patients (6%) were on amiodarone and 6 patients (3%) were on sotalol. After 1 year of SGLT2i therapy, a non‐significant increase in AAD prescription was detected (amiodarone 9.6% vs 6%, *P* value 0.190; sotalol 3% vs. 6%, *P* value 0.147). None of the patients undergone AA or VA ablation procedure during the study period.

**Table 1:** Study population characteristics.

Variable	Total population (*n* = 198)
Age (years)	72.5 (65;78)
Male sex (*n*, %)	171 (86.4%)
HTN (*n*, %)	144 (73%)
DM (*n*, %)	75 (38%)
Dyslipidaemia (*n*, %)	132 (66.7%)
COPD (*n*, %)	24 (12.1%)
CKD (*n*, %)	138 (70%)
Smoking (*n*, %)	105 (53%)
Previous TIA/stroke (*n*, %)	6 (3%)
HF aetiology	
Ischaemic	123 (62.2%)
Non‐ischaemic	57 (28.8%)
Valvular	9 (4.5%)
Inflammatory	9 (4.5%)
AF/AFL history (*n*, %)	87 (44%)
ACEIs (*n*, %)	30 (15.2%)
ARBs (*n*, %)	9 (4.5%)
ARNIs (*n*, %)	141 (71.2%)
Beta‐blockers (*n*, %)	180 (90.9%)
Dapagliflozin (*n*, %)	138 (69.7%)
Empagliflozin (*n*, %)	60 (30.3%)
MRAs (*n*, %)	135 (68.2%)
Loop diuretics (*n*, %)	126 (63.6%)
Amiodarone (*n*, %)	12 (6%)
Sotalol (*n*, %)	6 (3%)
Ivabradin (*n*, %)	5 (2.5%)
Digoxin (n, %)	8 (4%)
NYHA class (median)	2 (2;3)
LVEF (%)	30 (22.5;35)
CRT‐D	69 (34.8%)
Creatinine (mg/dL)	1.1 (0.96;1.5)
eGFR (mL/min/mq)	56.5 (42.9;76.5)
NT‐pro‐BNP (pg/mL)	497 (236;2442.3)

Abbreviations: ACEI, angiotensin‐converting enzyme inhibitor; AF, atrial fibrillation; AFL, atrial flutter; ARB, angiotensin receptor blocker; ARNI, angiotensin receptor‐neprilysin inhibitor; CRT‐D, cardiac resynchronization therapy defibrillator; CKD, chronic kidney disease; COPD, chronic obstructive pulmonary disease; CVD, cardiovascular disease; DM, diabetes mellitus; eGFR, estimated glomerular filtration rate; HF, heart failure; HFH, HF‐related hospitalization; HTN, hypertension; IVS, interventricular septum; LVEDD, Left ventricular end‐diastolic diameter; LVEF, left ventricular ejection fraction; MRA, mineralocorticoid receptor antagonist; NT‐pro‐BNP, N‐Terminal Pro‐B‐type Natriuretic Peptide; NYHA, New York Heart Association; PW= posterior wall; TAPSE, tricuspid annular plane systolic excursion; TIA, transient ischaemic attack.

**Table 2:** Cardiovascular medication prescription 1 year before and after SGLT2i therapy.

Variable	Before SGLT2i (*n* = 198)	After SGLT2i (*n* = 198)	*P* value
ACEIs (*n*, %)	30 (15.2%)	29 (14.6%)	0.887
ARBs (*n*, %)	9 (4.5%)	7 (3.5%)	0.609
ARNIs (*n*, %)	141 (71.2%)	145 (73.2%)	0.653
Beta‐blockers (*n*, %)	180 (90.9%)	178 (89.9%)	0.732
MRAs (*n*, %)	135 (68.2%)	130 (65.7%)	0.593
Loop Diuretics (*n*, %)	126 (63.6%)	112 (56.6%)	0.150
Amiodarone (*n*, %)	12 (6%)	20 (9.6%)	0.190
Sotalol (*n*, %)	6 (3%)	12 (6%)	0.147
Ivabradin (*n*, %)	5 (2.5%)	7 (3.5%)	0.557
Digoxin (*n*, %)	8 (4%)	4 (2%)	0.241

Abbreviations: ACEI, angiotensin‐converting enzyme inhibitor; ARB, angiotensin receptor blocker; ARNI, angiotensin receptor‐neprilysin inhibitor; MRA, mineralocorticoid receptor antagonist; SGLT2i, sodium glucose cotransporter 2 inhibitor.

### Primary outcomes

**Figure 2:**
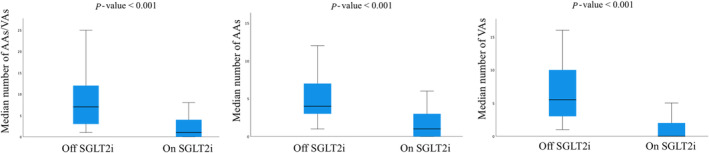
Box plots showing the rate of arrhythmic episodes before and after SGLT2i therapy initiation in patients with baseline arrhythmic events. AA, atrial arrhythmia; SGLT2i, sodium glucose cotransporter 2 inhibitor; VA, ventricular arrhythmia.

In patients with baseline arrhythmias, 1353 arrhythmic events were recorded in the year before SGLT2i therapy prescription and 354 events were detected in the year after SGLT2i initiation, with a 73.8% reduction in events number after therapy initiation (*P* value < 0.001). As shown in *Table* 3 and *Figure* 2, the median number of any AA/VA event was significantly reduced from 7 [3;12] off SGLT2i therapy to 1 [0;4] on SGLT2i therapy (*P* value < 0.001) among 135 patients with any arrhythmic event recorded at remote monitoring at baseline. On the same line, AA events were significantly reduced after SGLT2i therapy initiation from a median number of 4 [3;7] to 1 [0;3] (*P* value < 0.001). All the subtypes of AAs significantly decreased with gliflozin therapy, in particular AF events were reduced from 306 to 107 in 57 patients with at least one AF event during the year before therapy initiation (*Table* S1), from a median number of 4 [3;8] to 0 [0;3]. Among 102 patients with any VA at baseline, the median number of VA events significantly decreased from 5.5 [3;10] to 0 [0;2] (*P* value < 0.001). Larger reductions were recorded for NSVT that decreased from 4 [2;8.75] to 0 [0;2] (*P* value < 0.001) and for SVT that were reduced from 3 [2;4] to 0 [0;1] (*P* value < 0.001).

**Table 3:** Median number of arrhythmias pre‐ and post‐SGLT2i therapy in patients with baseline arrhythmic events.

	Off SGLT2i therapy	On SGLT2i Therapy	*P* value
Patients with any AA/VA at baseline (*n* = 135)			
Any AA/VA events (median)	7 (3;12)	1 (0;4)	<0.001
Patients with AAs (*n* = 87) at baseline			
Atrial events (median)	4 (3;7)	1 (0;3)	<0.001
Patients with AF (*n* = 57) at baseline			
AF events (median)	4 (3;8)	0 (0;3)	<0.001
Patients with AFL (*n* = 6) at baseline			
AFL events (median)	2(2;2)	0 (0;0)	0.002
Patients with AT (*n* = 36) at baseline			
AT events (median)	3 (2.25;4.75)	0 (0;1)	<0.001
Patients with VA (*n* = 102) at baseline			
VA events (median)	5.5 (3;10)	0 (0;2)	<0.001
Patients with NSVT (*n* = 96) at baseline			
NSVT events (median)	4 (2;8,75)	0 (0;2)	<0.001
Patients with SVT (*n* = 30) at baseline			
SVT events (median)	3 (2;4)	0 (0;1)	<0.001
Patients with VF (*n* = 18) at baseline			
VF events (median)	1.5 (1;3)	0 (0;0)	0.002
Patients with VA therapy (*n* = 18) at baseline			
VA therapy (median)	1.5 (1;3)	1 (1;1)	0.052

Abbreviations: AA, atrial arrhythmia; AF, atrial fibrillation; AFL, atrial flutter; AT, atrial tachycardia; NSVT, non‐sustained ventricular tachycardia; SGLT2i, sodium glucose cotransporter 2 inhibitor; SVT, sustained ventricular tachycardia; VA, ventricular arrhythmia; VF, ventricular fibrillation.

### Secondary outcomes

In total population, 1837 episodes were recorded during the study period going from 1 year before and 1 year after SGLT2i therapy starting date for each patient. After excluding 79 non‐arrhythmic episodes, 1758 episodes were analysed. The number of patients with any AA/VA, with AA and with VA events during the year after SGLT2i therapy initiation was significantly lower than during the year before SGLT2i prescription [102 vs. 135 (*P* value < 0.001), 54 vs. 87 (*P* value < 0.001), 60 vs. 102 (<0.001), respectively]. Furthermore, after SGLT2i therapy initiation, the median number of total arrhythmic episodes significantly decreased from a median of 4 [0;9] to 1 [0;3] (*P* value < 0.001). On the same line, AAs significantly decreased from a median of 2 [1;3] to 0 [0;1] episodes (*P* value < 0.001), and VAs were reduced from a median of 2 [1;2] to 0 [0;1] (*P* value < 0.001) after SGLT2i therapy initiation. The results are graphically represented in *Figure* S1. As shown in *Table* 4, after SGLT2i prescription, there was a significant reduction of the number of patients and number of arrhythmic episodes for all kinds of AA and VA, but AT. Indeed, only a non‐significant reduction in terms of median number of AT after gliflozin therapy initiation was recorded (*P* value 0.060). After SGLT2i therapy prescription, a significant reduction in total arrhythmic events, AAs and VAs was recorded despite the baseline HF therapy (Tables S2 and S3), whereas no significant differences in terms of arrhythmic events before and after gliflozin initiation were recorded in patients taking AADs at baseline or adding AADs during the study period (Tables S4–S7). As shown in Table S8, the significant reduction of arrhythmic events was confirmed when considering patients off AADs during the study period (*n* = 166).

**Table 4:** Secondary outcomes of the study in overall population.

Outcomes	Off SGLT2i therapy	On SGLT2i therapy	*P* value
Patients with any AA/VA	135	102	<0.001
Any AA/VA events (*n*)	1,353	405	
Any AA/VA events (median)	4 (0;9)	1 (0;3)	<0.001
Patients with AAs (*n*)	87	54	<0.001
Atrial events (*n*)	519	195	
Atrial events (median)	2 (1;3)	0 (0;1)	<0.001
Patients with AF (*n*)	57	39	0.035
AF events (*n*)	306	126	
AF events (median)	0 (0;3)	0 (0;0)	0.010
Patients with AFL (*n*)	6	0	0.014
AFL events (*n*)	12	0	
AFL events (median)	0 (0;0)	0 (0;0)	0.014
Patients with AT (*n*)	36	18	<0.001
AT events (*n*)	201	69	
AT events (median)	0 (0;0)	0 (0;0)	0.060
Patients with VA (*n*)	102	60	<0.001
VA events (*n*)	834	210	
VA events (median)	2 (1;2)	0 (0;1)	<0.001
Patients with NSVT (*n*)	96	45	<0.001
NSVT events (*n*)	660	180	
NSVT events (median)	0 (0;4)	0 (0;0)	<0.001
Patients with SVT (*n*)	30	12	0.003
SVT events (*n*)	102	12	
SVT events (median)	0 (0;0)	0 (0;0)	0.002
Patients with VF (*n*)	18	6	0.011
VF events (*n*)	36	12	
VF events (median)	0 (0;0)	0 (0;0)	0.010
Patients with VA therapy (*n*)	18	6	<0.001
VA therapy events (*n*)	36	6	
VA therapy (median)	0 (0;0)	0 (0;0)	0.010

Abbreviations: AA, atrial arrhythmia; AF, atrial fibrillation; AFL, atrial flutter; AT, atrial tachycardia; NSVT, non‐sustained ventricular tachycardia; SGLT2i, sodium glucose cotransporter 2 inhibitor; SVT, sustained ventricular tachycardia; VA, ventricular arrhythmia; VF, ventricular fibrillation.

The results of subgroup analyses are shown in Tables S9–S14. In general, the median number of any AA/VA significantly decreased during the year after SGLT2i prescription in all the subgroups. When considering AF, the number of patients with AF events and the median number of AF events were significantly decreased only in patients without diabetes and in patients with CRT, whereas a non‐significant reduction was observed in the other analysed subgroups. Patients with ischaemic cardiomyopathy and without CRT showed a significant reduction in SVT, VF and device ATP/shock after SGLT2i therapy introduction, as shown in Tables S11 and S13.

## Discussion

In the current analysis, the introduction of SGLT2i therapy was associated with a significant reduction of AAs and VAs in HFrEF carriers of ICD/CRT‐D followed by remote monitoring. The main findings of the study are as follows:

- In HFrEF patients with arrhythmic events off SGLT2i therapy, SGLT2i therapy prescription resulted in a significant 73.8% reduction in absolute number of arrhythmias and a reduction of the median number of arrhythmic events during the year after SGLT2i therapy initiation as compared with the year before SGLT2i prescription;
- In overall population, after SGLT2i therapy initiation, the median number of total arrhythmic episodes significantly decreased from a median of 4 [0;9] to 1 [0;3] (*P* value < 0.001), AAs significantly decreased from a median of 2 [1;3] to 0 [0;1] episodes (*P* value < 0.001) and VAs were reduced from a median of 2 [1;2] to 0 [0;1] (*P* value < 0.001);
- The median number of any AA/VA, VAs and NSVT significantly decreased during the year after SGLT2i prescription in all the subgroups, whereas the median number of AA events significantly decreased in all subgroups but patients with diabetes [0 (0;2) vs. 0 (0;1), *P* value 0.336];
- Only the subgroups of patients with ischaemic cardiomyopathy and without CRT showed a significant reduction in SVT, VF and device ATP/shock after SGLT2i therapy introduction.

HFrEF patients represent a category at high risk of adverse CV outcomes, including HFHs and CV death, and cardiac arrhythmias are known precipitating factors. Indeed, AF impacts on mortality and morbidity in HFrEF patients, leading to deterioration in quality of life, mental health, HFHs and CV death.^2^ Unfortunately, AF and HF share the same risk factors and a similar pathophysiology, so that AF may lead to HF and HF is often complicated by AF onset. According to the Framingham Heart Study, almost 40% of individuals with either AF or HF will develop the other condition and in individuals with HF, subsequent development of AF is associated with increased all‐cause mortality.^2^ VAs are common in HFrEF and are the leading cause of SCD in this population, strongly associated with poor prognosis. Even NSVT demonstrated to be associated with appropriate ICD therapy and HFHs in patients with primary prevention ICD (without previous appropriate ICD therapy).^13^ In HFrEF population, current guidelines suggest ICD implantation in primary prevention to reduce SCD rates in HFrEF patients.^4^ Notably, HFrEF carriers of ICD that receive an appropriate ICD shock have almost 6‐fold increased risk of death from any cause at follow‐up.^14^ Hence, in patients with HFrEF the reduction of atrial and ventricular arrhythmic burden is pivotal and should be pursued to increase patients' prognosis. HF drugs such as ACEi/ARBs, MRAs, beta‐blockers showed to reduce both AAs and VAs in HFrEF patients.^15^ Conversely, SGLT2i effect on arrhythmias has not been fully elucidated yet. To date, only a post‐hoc analysis of the DECLARE‐TIMI 58 trial showed reduction of AF incidence and recurrence in patients treated with dapagliflozin as compared with control arm.^16^ In a meta‐analysis, gliflozins have demonstrated to reduce AF occurrence, but the risk of VAs was not different among SGLT2i and control.^9^, ^10^ In a network meta‐analysis, Mariani et al.^17^ showed an 11% decrease of AF occurrence in patients treated with gliflozins, with dapagliflozin showing the largest risk reduction of AF at follow‐up. However, in the main RCTs on SGLT2i, arrhythmic events were reported as serious adverse events and not as primary endpoint event. As a result, only symptomatic arrhythmic episodes were reported in RCTs, possibly leading to underreporting bias. Conversely, our real‐world study overcomes the limitations of previously published RCTs and meta‐analysis, reporting every arrhythmic event as detected at ICD/CRT‐D interrogation, even the asymptomatic episodes, thus providing the actual arrhythmic burden on and off SGLT2i therapy. We reported that the atrial and ventricular arrhythmic burden in HFrEF carriers of ICD/CRT‐D is significantly reduced after SGLT2i therapy prescription. The rapid diuretic/natriuretic effect, with reduction of cardiac filling pressure, may not solely explain the antiarrhythmic effect of SGLT2i drugs. Indeed, significant reductions in AAs were seen in meta‐analysis only including RCTs with a follow‐up >1 year.^9^, ^10^ Moreover, Ziyrek et al.^18^ found that SGLT2i led to decreases in P wave dispersion, in left‐ and right‐sided intra‐atrial electromechanical delay and in interatrial electromechanical delay, suggesting that the electrical remodelling may be one of the mechanisms of the decrease in the frequency of AF with the use of SGLT2i, but the effects on atrial electromechanics became evident only after 6 months therapy. Hence, several other mechanisms have been advocated to explain arrhythmias reduction associated with SGLT2i therapy, such as restoration of calcium and sodium homeostasis, antioxidant and anti‐inflammatory effect and reverse cardiac remodelling.^15^

We reported a significant reduction in total AA episodes and in all the different subtypes of AA except for AT in the overall population, whereas the median number of AA events significantly decreased in all subgroups but patients with diabetes [0 (0;2) vs. 0 (0;1), *P* value 0.336]. When considering AF/AHRE, only the subgroup of patients without diabetes showed a significant reduction in median number of events (*P* value 0.007). Our report of significant AA reduction after SGLT2i therapy initiation confirms the findings of meta‐analysis reporting reduction of AA incidence with SGLT2i as compared with control.^9^, ^10^ Similarly to our study, Younis et al.^19^ evaluated the effect of SGLT2i on arrhythmic events in patients with CIED, reporting that the use of SGLT2i was independently associated with a significant 22% reduction in the risk of AAs and 22% significant reduction of AA/VA, without a significant benefit in terms of VA risk reduction. Moreover, AAs were significantly reduced in HF subgroup of patients but not among patients with diabetes. Interestingly, in line with Younis et al.^19^ findings, we did not report a significant reduction of total AA and AF/AHRE in patients with diabetes. This result may be related to the robust association between diabetes, AAs and diabetic cardiomyopathy that make difficult AA control in diabetic patients. Huxley et al.^20^ showed in a meta‐analysis of cohort studies that diabetes is correlated with a 40% increased risk of AF. Inflammation, oxidative stress and glycaemic fluctuations lead to atrial fibrosis and dilation with increased electromechanical delay and increased vulnerability to AAs.^21^ Concerning VAs, we found a significant reduction in the number of patients experiencing VAs and in the median number of VAs after SGLT2i prescription in the overall population. These findings have never been reported in previous studies because meta‐analysis and the study by Younis et al. failed to demonstrate a reduction of VA burden with SGLT2i therapy.^9^, ^10^, ^19^ Our significant results may be related to the peculiar population included in our study, that enrolled all patients with HFrEF that are at higher risk of VAs as compared with the general population. The meta‐analysis by Fernandes et al.^9^ failed to show a significant reduction in the risk of incident VAs associated with SGLT2i therapy but only one of the included RCTs enrolled HFrEF patients. Younis et al.^19^ reported a non‐significant 8% reduction of VA risk in CIED carriers, but HF was only present in 1/3 of patients. Alongside enrolled population differences, our results may also be related to the inclusion among VAs of NSVTs, that were the most common VA detected, and that were not computed in previous studies. However, in view of the prognostic implication of NSVTs in HFrEF patients with ICD in primary prevention,^22^ the inclusion of NSVTs has a clinical rationale and may strengthen the results of our analysis. Eventually, we found a significant reduction of every type of VAs only in two subgroups of patients, those with ischaemic cardiomyopathy and those without CRT. Conversely the median number of SVT, VF and device therapy did not differ on and off SGLT2i therapy among patients with non‐ischaemic cardiomyopathy and in patients with CRT. These findings may be related with the higher propensity to experience VA and SCD reported for ischaemic cardiomyopathy as compared with non‐ischaemic cardiomyopathy and with the reduction of VA associated with CRT‐D as compared with ICD in HFrEF patients receiving the device in primary prevention of SCD.^4^, ^23^ However, the non‐significant reduction of SVT, VF and device therapy in these subgroups may also be related to the small number of patients with non‐ischaemic cardiomyopathy (*n* = 75) and with CRT (*n* = 69) included in the study. Hence, our findings should be taken with caution until larger, well‐designed RCTs will clarify the antiarrhythmic effect of SGLT2i in these subgroups of patients.

## Limitations

Our study presents some limitations. To begin with, the study has a retrospective, non‐randomized design; thus, the observed outcomes may be affected by potential confounding factors. However, we excluded patients who discontinued one of the four HF drug therapy pillars after the SGLT2i therapy initiation so that no difference in terms of HF drug therapy prescription rate is present before and after SGLT2i therapy initiation (*Table* 2). As show in *Table* 2, the number of patients on AAD was comparable during the year before and after SGLT2i prescription and none of the patients undergone AA or VA ablation procedure during the study period, thus reducing the risk of confounding factors. Moreover, the analysis including patients without AAD therapy during the study period confirmed the significant reduction of arrhythmic events found in the main analysis (Table S8). Second, the study included a small number of patients, limiting the analysis of SGLT2i prescription effect in subgroups of patients. We included a highly selected population (HFrEF patients with ICD/CRT‐D followed by remote monitoring) so that our results may not be extended to the general population. However, the included population presents the highest risk of AAs and VAs, and the expected high rates of arrhythmias together with the reliable methodology to detect endpoints (device interrogation at remote monitoring) strengthen the validity of our analysis. Third, we programmed detection and therapy windows of CIEDs according to manufacturer‐specific recommendations, leading in differences of programming among different brands; however, this is not an actual bias because each case serves as its own control, i.e. the study is self‐matched. Fourth, although HF OMT was prescribed at the maximum tolerated dose in all patients, unless contraindicated or not tolerated, we expect some differences in HF drug dosages among patients. Unfortunately, drug dosages for each HF medication were not available but, as per‐protocol definition, patients who discontinued one of the four pillars of HF drug therapy were excluded from the analysis, so that no difference in terms of HF OMT prescription was present before and after SGLT2i prescription (*Table* 2). Fifth, our study was not designed to unravel the mechanistic insights of antiarrhythmic effect associated with SGLT2i use, but our results may fuel research to shed light on this issue, especially in some subgroup of patients. Eventually, 1 year of follow‐up after SGLT2i therapy prescription may be short. However, several meta‐analyses reported a significant anti‐arrhythmic effect of SGLT2i therapy in study with at least 1 year of follow‐up, whereas studies with a follow‐up <1 year did not find any SGLT2i effect on arrhythmia occurrence.^9^, ^10^

## Conclusions

In HFrEF carriers of ICD/CRT‐D followed by remote monitoring, the use of SGLT2i was associated with significant reduction in terms of AA and VA events. Large, well‐designed RCTs are needed to confirm our results and to further clarify the anti‐arrhythmic potential of SGLT2i pharmacological class.

## Conflict of interest statement

The authors declare no conflict of interest.

## Funding

This research did not receive any specific grant from founding agencies in the public, commercial or not‐for‐profit sectors.

## Supporting information


**Table S1.** Number of arrhythmias pre‐ and post‐SGLT2i therapy in patients with baseline arrhythmic events.
**Table S2.** Total, atrial and ventricular events in patients taking beta‐blockers, ACE‐I/ARB/ARNI and MRA during the study period.
**Table S3.** Total, atrial and ventricular events in patients not taking beta‐blockers, ACE‐I/ARB/ARNI and MRA during the study period.
**Table S4.** Total, atrial and ventricular events in patients taking amiodarone during the study period.
**Table S5.** Total, atrial and ventricular events in patients taking sotalol during the study period.
**Table S6.** Total, atrial and ventricular events in patients introducing amiodarone after SGLT2i prescription.
**Table S7.** Total, atrial and ventricular events in patients introducing sotalol after SGLT2i prescription.
**Table S8.** Total, atrial and ventricular events in patients off AADs during the study period.
**Table S9.** Subgroup analysis: primary and secondary outcomes in patients without diabetes mellitus.
**Table S10.** Subgroup analysis: primary and secondary outcomes in patients with diabetes mellitus.
**Table S11.** Subgroup analysis: primary and secondary outcomes in patients with ischaemic cardiomyopathy.
**Table S12.** Subgroup analysis: primary and secondary outcomes in patients with non‐ischaemic cardiomyopathy.
**Table S13.** Subgroup analysis: primary and secondary outcomes in patients without CRT.
**Table S14.** Subgroup analysis: primary and secondary outcomes in patients with CRT.

**Figure S1.** Bar graphs and box plots showing the rate of arrhythmic episodes before and after SGLT2i therapy initiation.
