# Supplementary material for: SGLT2i reduce arrhythmic events in heart failure patients with cardiac implantable electronic devices
Source: ESC Heart Fail. 2025 Feb 7;12(3):2125–33. doi: 10.1002/ehf2.15223 (PMC12055389; doi:10.1002/ehf2.15223)

**Table A.1 Number of arrhythmias pre- and post-SGLT2i therapy in patients with baseline arrhythmic events.**

|  | **Off SGLT2i therapy** | **On SGLT2i Therapy** |  |
| --- | --- | --- | --- |
|  | |  |  |
| **Any AA/VA events (n)** | 1353 | 354 |  |
| **Atrial Events (n)** | 519 | 168 |  |
| **AF events (n)** | 306 | 107 |  |
| **AFL events (n)** | 12 | 0 |  |
| **AT events (n)** | 201 | 61 |  |
| **VA events (n)** | 834 | 186 |  |
| **NSVT events (n)** | 660 | 171 |  |
| **SVT events (n)** | 102 | 6 |  |
| **VF events (n)** | 36 | 3 |  |
| **Therapy events (n)** | 36 | 6 |  |

**AA = atrial arrhythmia; AF = atrial fibrillation; AFL = atrial flutter; AT = atrial tachycardia; NSVT = non-sustained ventricular tachycardia; SGLT2i = sodium glucose cotransporter 2 inhibitor; SVT = sustained ventricular tachycardia; VA = ventricular arrhythmia; VF = ventricular fibrillation.**

**Table A.2. Total, atrial and ventricular events in patients taking beta-blockers, ACE-I/ARB/ARNI and MRA during the study period.**

| **Outcomes** | **OMT without SGLT2i therapy (n=111)** | **OMT with SGLT2i Therapy (n=111)** | **p-value** |
| --- | --- | --- | --- |
|  |  |  |  |
| **Any AA/VA events (n)** | **654** | **201** |  |
| **Any AA/VA events (median)** | **4 (0;9)** | **1 (0;2)** | **< 0.001** |
| **Atrial events (n)** | **258** | **117** |  |
| **Atrial events (median)** | **0 (0;4)** | **0 (0;1)** | **< 0.001** |
| **VA events (n)** | **396** | **84** |  |
| **VA events (median)** | **1 (1;4)** | **0 (0;1)** | **< 0.001** |

**AA = atrial arrhythmia; ACE-I = angiotensin-converting enzyme inhibitors; ARB = angiotensin receptor blockers; ARNI = angiotensin receptor-neprilysin inhibitor; MRA = mineralcorticoid receptor antagonists; SGLT2i = sodium glucose cotransporter 2 inhibitor; VA = ventricular arrhythmia**

**Table A.3. Total, atrial and ventricular events in patients not taking beta-blockers, ACE-I/ARB/ARNI and MRA during the study period.**

| **Outcomes** | **No OMT without SGLT2i therapy (n=87)** | **No OMT with SGLT2i Therapy (n=87)** | **p-value** |
| --- | --- | --- | --- |
|  |  |  |  |
| **Any AA/VA events (n)** | 699 | 204 |  |
| **Any AA/VA events (median)** | 3 (1;12) | 0 (0;3) | < 0.001 |
| **Atrial events (n)** | 261 | 78 |  |
| **Atrial events (median)** | 0 (0;3) | 0 (0;0) | 0.007 |
| **VA events (n)** | 438 | 126 |  |
| **VA events (median)** | 1 (0;8) | 0 (0;1) | < 0.001 |

AA = atrial arrhythmia; ACE-I = angiotensin-converting enzyme inhibitors; ARB = angiotensin receptor blockers; ARNI = angiotensin receptor-neprilysin inhibitor; MRA = mineralcorticoid receptor antagonists; SGLT2i = sodium glucose cotransporter 2 inhibitor; VA = ventricular arrhythmia

**Table A.4. Total, atrial and ventricular events in patients taking amiodarone during the study period**

| **Outcomes** | **On Amiodarone before SGLT2i prescription (n = 12)** | **On Amiodarone after SGLT2i prescription (n = 20)** | **p-value** |
| --- | --- | --- | --- |
|  |  |  |  |
| **Any AA/VA events (n)** | 12 | 9 |  |
| **Any AA/VA events (median)** | 0 (0;3) | 0 (0;2.25) | 0.799 |
| **Atrial events (n)** | 12 | 9 |  |
| **Atrial events (median)** | 0 (0;3) | 0 (0;2.25) | 0.799 |
| **VA events (n)** | 0 | 0 |  |
| **VA events (median)** | 0 | 0 | 1 |

AA = atrial arrhythmia; SGLT2i = sodium glucose cotransporter 2 inhibitor; VA = ventricular arrhythmia

**Table A.5. Total, atrial and ventricular events in patients taking sotalol during the study period**

| **Outcomes** | **On Sotalol before SGLT2i prescription (n = 6)** | **On Sotalol after SGLT2i prescription (n = 6)** | **p-value** |
| --- | --- | --- | --- |
|  |  |  |  |
| **Any AA/VA events (n)** | 12 | 9 |  |
| **Any AA/VA events (median)** | 2 (2;2) | 1 (0;3.25) | 0.394 |
| **Atrial events (n)** | 12 | 9 |  |
| **Atrial events (median)** | 2 (2;2) | 1 (0;3.25) | 0.394 |
| **VA events (n)** | 0 | 0 |  |
| **VA events (median)** | **0** | **0** | **1** |

AA = atrial arrhythmia; SGLT2i = sodium glucose cotransporter 2 inhibitor; VA = ventricular arrhythmia

**Table A.6. Total, atrial and ventricular events in patients introducing amiodarone after SGLT2i prescription**

| **Outcomes** | **Off Amiodarone before SGLT2i prescription (n = 8)** | **On Amiodarone after SGLT2i prescription (n = 8)** | **p-value** |
| --- | --- | --- | --- |
|  |  |  |  |
| **Any AA/VA events (n)** | 10 | 17 |  |
| **Any AA/VA events (median)** | 0.5 (0;2) | 1 (1;1.75) | 0.505 |
| **Atrial events (n)** | 4 | 3 |  |
| **Atrial events (median)** | 0 (0;1) | 0 (0;1) | 0.878 |
| **VA events (n)** | 6 | 14 |  |
| **VA events (median)** | 0 (0;1.5) | 1 (0.25;1) | 0.234 |

AA = atrial arrhythmia; SGLT2i = sodium glucose cotransporter 2 inhibitor; VA = ventricular arrhythmia

**Table A.7. Total, atrial and ventricular events in patients introducing sotalol after SGLT2i prescription**

| **Outcomes** | **Off Sotalol before SGLT2i prescription (n = 6)** | **On Sotalol after SGLT2i prescription (n = 6)** | **p-value** |
| --- | --- | --- | --- |
|  |  |  |  |
| **Any AA/VA events (n)** | 10 | 14 |  |
| **Any AA/VA events (median)** | 1.5 (0;2.75) | 1 (0;4) | 0.937 |
| **Atrial events (n)** | 4 | 2 |  |
| **Atrial events (median)** | 0.5 (0;1.25) | 0 (0;1) | 0.589 |
| **VA events (n)** | 6 | 12 |  |
| **VA events (median)** | 0 (0;2.5) | 1 (0;3) | 0.589 |

AA = atrial arrhythmia; SGLT2i = sodium glucose cotransporter 2 inhibitor; VA = ventricular arrhythmia

**Table A.8. Total, atrial and ventricular events in patients off AADs during the study period**

| **Outcomes** | **Off AADs before SGLT2i prescription (n = 166)** | **Off AADs after SGLT2i prescription (n = 166)** | **p-value** |
| --- | --- | --- | --- |
|  |  |  |  |
| **Any AA/VA events (n)** | 1309 | 356 |  |
| **Any AA/VA events (median)** | 4 (2;10) | 1 (0;3) | < 0.001 |
| **Atrial events (n)** | 487 | 172 |  |
| **Atrial events (median)** | 1 (0;4) | 0 (0;1) | < 0.001 |
| **AF events (n)** | 277 | 103 |  |
| **AF events (median)** | 0 (0;3) | 0 (0;0) | 0.003 |
| **AFL events (n)** | 12 | 0 |  |
| **AFL events (median)** | 0 (0;0) | 0 (0;0) | 0.014 |
| **AT events (n)** | 198 | 69 |  |
| **AT events (median)** | 0 (0;0) | 0 (0;0) | 0.07 |
| **VA events (n)** | 822 | 184 |  |
| **VA events (median)** | 2 (0;8) | 0 (0;1) | < 0.001 |
| **NSVT events (n)** | 648 | 161 |  |
| **NSVT events (median)** | 1 (0;6) | 0 (0;0.25) | <0.001 |
| **SVT events (n)** | 102 | 11 |  |
| **SVT events (median)** | 0 (0;0) | 0 (0;0) | <0.001 |
| **VF events (n)** | 36 | 6 |  |
| **VF events (median)** | 0 (0;0) | 0 (0;0) | 0.002 |
| **VA therapy events (n)** | 36 | 6 |  |
| **VA therapy (median)** | 0 (0;0) | 0 (0;0) | 0.009 |

AA = atrial arrhythmia; AAD = antiarrhythmic drug; AF = atrial fibrillation; AFL = atrial flutter; AT = atrial tachycardia; NSVT = non-sustained ventricular tachycardia; SGLT2i = sodium glucose cotransporter 2 inhibitor; SVT = sustained ventricular tachycardia; VA = ventricular arrhythmia; VF = ventricular fibrillation.

**Table A.9. Subgroup analysis: primary and secondary outcomes in patients without diabetes mellitus.**

| **Patients without diabetes (n=123)** | **Off SGLT2i therapy** | **On SGLT2i Therapy** | **p-value** |
| --- | --- | --- | --- |
| Patients with any AA/VA | 81 | 66 | 0.051 |
| Any AA/VA events (n) | 917 | 265 |  |
| Any AA/VA events (median) | 4 (0;9) | 1 (0;3) | < 0.001 |
| Patients with AAs (n) | 60 | 30 | < 0.001 |
| Atrial events (n) | 353 | 118 |  |
| Atrial events (median) | 0.5 (0;4) | 0 (0;1) | < 0.001 |
| Patients with AF (n) | 39 | 21 | 0.008 |
| AF events (n) | 179 | 61 |  |
| AF events (median) | 0 (0;3) | 0 (0;0) | 0.007 |
| Patients with AFL (n) | 3 | 0 | 0.247 |
| AFL events (n) | 6 | 0 |  |
| AFL events (median) | 0 (0;0) | 0 (0;0) | 0.082 |
| Patients with AT (n) | 24 | 12 | 0.030 |
| AT events (n) | 168 | 57 |  |
| AT events (median) | 0 (0;0) | 0 (0;0) | 0.095 |
| Patients with VA (n) | 60 | 42 | 0.020 |
| VA events (n) | 564 | 147 |  |
| VA events (median) | 0 (0;6) | 0 (0;1) | 0.001 |
| Patients with NSVT (n) | 60 | 33 | < 0.001 |
| NSVT events (n) | 471 | 129 |  |
| NSVT events (median) | 0 (0;4) | 0 (0;0) | <0.001 |
| Patients with SVT (n) | 15 | 9 | 0.197 |
| SVT events (n) | 63 | 9 |  |
| SVT events (median) | 0 (0;0) | 0 (0;0) | 0.141 |
| Patients with VF (n) | 9 | 3 | 0.076 |
| VF events (n) | 15 | 9 |  |
| VF events (median) | 0 (0;0) | 0 (0;0) | 0.084 |
| Patients with VA therapy (n) | 9 | 0 | 0.003 |
| VA therapy events (n) | 15 | 0 |  |
| VA therapy (median) | 0 (0;0) | 0 (0;0) | 0.002 |

AA = atrial arrhythmia; AF = atrial fibrillation; AFL = atrial flutter; AT = atrial tachycardia; NSVT = non-sustained ventricular tachycardia; SGLT2i = sodium glucose cotransporter 2 inhibitor; SVT = sustained ventricular tachycardia; VA = ventricular arrhythmia; VF = ventricular fibrillation.

**Table A.10. Subgroup analysis: primary and secondary outcomes in patients with diabetes mellitus.**

| **Patients with diabetes (n=75)** | **Off SGLT2i therapy** | **On SGLT2i Therapy** | **p-value** |
| --- | --- | --- | --- |
| Patients with any AA/VA | 54 | 36 | 0.003 |
| Any AA/VA events (n) | 436 | 140 |  |
| Any AA/VA events (median) | 4 (1;10) | 0 (0;3) | < 0.001 |
| Patients with AAs (n) | 27 | 24 | 0.605 |
| Atrial events (n) | 166 | 77 |  |
| Atrial events (median) | 0 (0;2) | 0 (0;1) | 0.336 |
| Patients with AF (n) | 18 | 18 | 1 |
| AF events (n) | 127 | 65 |  |
| AF events (median) | 0 (0;0) | 0 (0;0) | 0.648 |
| Patients with AFL (n) | 3 | 0 | 0.245 |
| AFL events (n) | 6 | 0 |  |
| AFL events (median) | 0 (0;0) | 0 (0;0) | 0.081 |
| Patients with AT (n) | 12 | 6 | 0.132 |
| AT events (n) | 33 | 12 |  |
| AT events (median) | 0 (0;0) | 0 (0;0) | 0.369 |
| Patients with VA (n) | 42 | 18 | < 0.001 |
| VA events (n) | 270 | 63 |  |
| VA events (median) | 2 (0;6) | 0 (0;0) | < 0.001 |
| Patients with NSVT (n) | 36 | 12 | < 0.001 |
| NSVT events (n) | 189 | 51 |  |
| NSVT events (median) | 0 (0;3) | 0 (0;0) | < 0.001 |
| Patients with SVT (n) | 15 | 3 | 0.003 |
| SVT events (n) | 39 | 3 |  |
| SVT events (median) | 0 (0;0) | 0 (0;0) | 0.002 |
| Patients with VF (n) | 9 | 3 | 0.071 |
| VF events (n) | 21 | 3 |  |
| VF events (median) | 0 (0;0) | 0 (0;0) | 0.072 |
| Patients with VA therapy (n) | 9 | 6 | 0.414 |
| VA therapy events (n) | 21 | 6 |  |
| VA therapy (median) | 0 (0;0) | 0 (0;0) | 0.346 |

AA = atrial arrhythmia; AF = atrial fibrillation; AFL = atrial flutter; AT = atrial tachycardia; NSVT = non-sustained ventricular tachycardia; SGLT2i = sodium glucose cotransporter 2 inhibitor; SVT = sustained ventricular tachycardia; VA = ventricular arrhythmia; VF = ventricular fibrillation.

**Table A.11. Subgroup analysis: primary and secondary outcomes in patients with ischemic cardiomyopathy.**

| **Patients with ischemic cardiomyopathy (n=123)** | **Off SGLT2i therapy** | **On SGLT2i Therapy** | **p-value** |
| --- | --- | --- | --- |
| Patients with any AA/VA | 77 | 60 | 0.029 |
| Any AA/VA events (n) | 720 | 223 |  |
| Any AA/VA events (median) | 3 (0;9) | 0 (0;2) | < 0.001 |
| Patients with AAs (n) | 45 | 26 | 0.008 |
| Atrial events (n) | 299 | 85 |  |
| Atrial events (median) | 0 (0;3) | 0 (0;0) | 0.004 |
| Patients with AF (n) | 28 | 18 | 0.102 |
| AF events (n) | 149 | 51 |  |
| AF events (median) | 0 (0;0) | 0 (0;0) | 0.052 |
| Patients with AFL (n) | 4 | 0 | 0.122 |
| AFL events (n) | 8 | 0 |  |
| AFL events (median) | 0 (0;0) | 0 (0;0) | 0.044 |
| Patients with AT (n) | 19 | 9 | 0.045 |
| AT events (n) | 142 | 34 |  |
| AT events (median) | 0 (0;0) | 0 (0;0) | 0.200 |
| Patients with VA (n) | 61 | 37 | 0.002 |
| VA events (n) | 421 | 138 |  |
| VA events (median) | 0 (0;6) | 0 (0;1) | < 0.001 |
| Patients with NSVT (n) | 55 | 31 | 0.001 |
| NSVT events (n) | 316 | 126 |  |
| NSVT events (median) | 0 (0;3) | 0 (0;1) | < 0.001 |
| Patients with SVT (n) | 19 | 5 | 0.003 |
| SVT events (n) | 61 | 5 |  |
| SVT events (median) | 0 (0;0) | 0 (0;0) | 0.002 |
| Patients with VF (n) | 13 | 2 | 0.003 |
| VF events (n) | 22 | 4 |  |
| VF events (median) | 0 (0;0) | 0 (0;0) | 0.004 |
| Patients with VA therapy (n) | 13 | 3 | 0.010 |
| VA therapy events (n) | 22 | 3 |  |
| VA therapy (median) | 0 (0;0) | 0 (0;0) | 0.009 |

AA = atrial arrhythmia; AF = atrial fibrillation; AFL = atrial flutter; AT = atrial tachycardia; NSVT = non-sustained ventricular tachycardia; SGLT2i = sodium glucose cotransporter 2 inhibitor; SVT = sustained ventricular tachycardia; VA = ventricular arrhythmia; VF = ventricular fibrillation.

**Table A.12. Subgroup analysis: primary and secondary outcomes in patients with non-ischemic cardiomyopathy.**

|  |  |  |  |
| --- | --- | --- | --- |
| **Patients with non-ischemic cardiomyopathy (n=75)** | **Off SGLT2i therapy** | **On SGLT2i Therapy** | **p-value** |
| Patients with any AA/VA | 58 | 42 | 0.006 |
| Any AA/VA events (n) | 633 | 182 |  |
| Any AA/VA events (median) | 4 (2;12) | 1 (0;3) | < 0.001 |
| Patients with AAs (n) | 42 | 28 | 0.022 |
| Atrial events (n) | 220 | 110 |  |
| Atrial events (median) | 1 (0;4) | 0 (0;2) | 0.012 |
| Patients with AF (n) | 29 | 21 | 0.166 |
| AF events (n) | 157 | 75 |  |
| AF events (median) | 0 (0;4) | 0 (0;1) | 0.075 |
| Patients with AFL (n) | 2 | 0 | 0.155 |
| AFL events (n) | 4 | 0 |  |
| AFL events (median) | 0 (0;0) | 0 (0;0) | 0.156 |
| Patients with AT (n) | 17 | 9 | 0.084 |
| AT events (n) | 59 | 35 |  |
| AT events (median) | 0 (0;0) | 0 (0;0) | 0.173 |
| Patients with VA (n) | 41 | 23 | 0.003 |
| VA events (n) | 413 | 72 |  |
| VA events (median) | 1 (0;6) | 0 (0;1) | <0.001 |
| Patients with NSVT (n) | 41 | 14 | < 0.001 |
| NSVT events (n) | 344 | 54 |  |
| NSVT events (median) | 1 (0;5) | 0 (0;0) | <0.001 |
| Patients with SVT (n) | 11 | 7 | 0.315 |
| SVT events (n) | 41 | 7 |  |
| SVT events (median) | 0 (0;0) | 0 (0;0) | 0.209 |
| Patients with VF (n) | 5 | 4 | 1 |
| VF events (n) | 14 | 8 |  |
| VF events (median) | 0 (0;0) | 0 (0;0) | 0.705 |
| Patients with VA therapy (n) | 5 | 3 | 0.719 |
| VA therapy events (n) | 14 | 3 |  |
| VA therapy (median) | 0 (0;0) | 0 (0;0) | 0.443 |

AA = atrial arrhythmia; AF = atrial fibrillation; AFL = atrial flutter; AT = atrial tachycardia; NSVT = non-sustained ventricular tachycardia; SGLT2i = sodium glucose cotransporter 2 inhibitor; SVT = sustained ventricular tachycardia; VA = ventricular arrhythmia; VF = ventricular fibrillation.

**Table A.13. Subgroup analysis: primary and secondary outcomes in patients without CRT.**

|  |  |  |  |
| --- | --- | --- | --- |
| **Patients without CRT (n=129)** | **Off SGLT2i therapy** | **On SGLT2i Therapy** | **p-value** |
| Patients with any AA/VA | 84 | 72 | 0.127 |
| Any AA/VA events (n) | 888 | 336 |  |
| Any AA/VA events (median) | 4 (0;10) | 1 (0;4) | < 0.001 |
| Patients with AAs (n) | 54 | 39 | 0.052 |
| Atrial events (n) | 300 | 153 |  |
| Atrial events (median) | 0 (0;3) | 0 (0;1) | 0.020 |
| Patients with AF (n) | 36 | 30 | 0.392 |
| AF events (n) | 231 | 105 |  |
| AF events (median) | 0 (0;3) | 0 (0;0) | 0.164 |
| Patients with AFL (n) | 3 | 0 | 0.247 |
| AFL events (n) | 6 | 0 |  |
| AFL events (median) | 0 (0;0) | 0 (0;0) | 0.082 |
| Patients with AT (n) | 21 | 12 | 0.093 |
| AT events (n) | 63 | 48 |  |
| AT events (median) | 0 (0;0) | 0 (0;0) | 0.227 |
| Patients with VA (n) | 66 | 42 | 0.002 |
| VA events (n) | 588 | 183 |  |
| VA events (median) | 1 (0;7) | 0 (0;1) | < 0.001 |
| Patients with NSVT (n) | 60 | 33 | < 0.001 |
| NSVT events (n) | 507 | 162 |  |
| NSVT events (median) | 0 (0;6) | 0 (0;1) | < 0.001 |
| Patients with SVT (n) | 21 | 9 | 0.020 |
| SVT events (n) | 51 | 9 |  |
| SVT events (median) | 0 (0;0) | 0 (0;0) | 0.012 |
| Patients with VF (n) | 12 | 3 | 0.017 |
| VF events (n) | 15 | 9 |  |
| VF events (median) | 0 (0;0) | 0 (0;0) | 0.021 |
| Patients with VA therapy (n) | 12 | 3 | 0.017 |
| VA therapy events (n) | 15 | 3 |  |
| VA therapy (median) | 0 (0;0) | 0 (0;0) | 0.016 |

AA = atrial arrhythmia; AF = atrial fibrillation; AFL = atrial flutter; AT = atrial tachycardia; CRT = cardiac resynchronization therapy; NSVT = non-sustained ventricular tachycardia; SGLT2i = sodium glucose cotransporter 2 inhibitor; SVT = sustained ventricular tachycardia; VA = ventricular arrhythmia; VF = ventricular fibrillation.

**Table A.14. Subgroup analysis: primary and secondary outcomes in patients with CRT.**

|  |  |  |  |
| --- | --- | --- | --- |
| **Patients with CRT (n=69)** | **Off SGLT2i therapy** | **On SGLT2i Therapy** | **p-value** |
| Patients with any AA/VA | 51 | 30 | < 0.001 |
| Any AA/VA events (n) | 465 | 69 |  |
| Any AA/VA events (median) | 3 (2;9) | 0 (0;1) | < 0.001 |
| Patients with AAs (n) | 33 | 15 | 0.001 |
| Atrial events (n) | 219 | 42 |  |
| Atrial events (median) | 0 (0;4) | 0 (0;1) | < 0.001 |
| Patients with AF (n) | 21 | 9 | 0.013 |
| AF events (n) | 75 | 21 |  |
| AF events (median) | 0 (0;3) | 0 (0;0) | 0.008 |
| Patients with AFL (n) | 3 | 0 | 0.245 |
| AFL events (n) | 6 | 0 |  |
| AFL events (median) | 0 (0;0) | 0 (0;0) | 0.081 |
| Patients with AT (n) | 15 | 6 | 0.033 |
| AT events (n) | 138 | 21 |  |
| AT events (median) | 0 (0;0) | 0 (0;0) | 0.131 |
| Patients with VA (n) | 36 | 18 | 0.002 |
| VA events (n) | 246 | 27 |  |
| VA events (median) | 2 (0;3) | 0 (0;1) | < 0.001 |
| Patients with NSVT (n) | 36 | 12 | < 0.001 |
| NSVT events (n) | 153 | 18 |  |
| NSVT events (median) | 2 (0;3) | 0 (0;0) | < 0.001 |
| Patients with SVT (n) | 9 | 3 | 0.070 |
| SVT events (n) | 51 | 3 |  |
| SVT events (median) | 0 (0;0) | 0 (0;0) | 0.055 |
| Patients with VF (n) | 6 | 3 | 0.493 |
| VF events (n) | 21 | 3 |  |
| VF events (median) | 0 (0;0) | 0 (0;0) | 0.292 |
| Patients with VA therapy (n) | 6 | 3 | 0.493 |
| VA therapy events (n) | 21 | 3 |  |
| VA therapy (median) | 0 (0;0) | 0 (0;0) | 0.263 |

AA = atrial arrhythmia; AF = atrial fibrillation; AFL = atrial flutter; AT = atrial tachycardia; CRT = cardiac resynchronization therapy; NSVT = non-sustained ventricular tachycardia; SGLT2i = sodium glucose cotransporter 2 inhibitor; SVT = sustained ventricular tachycardia; VA = ventricular arrhythmia; VF = ventricular fibrillation.

**Figure A.1. Bar graphs and box plots showing the rate of arrhythmic episodes before and after SGLT2i therapy initiation.**

AA = atrial arrhythmia; SGLT2i = sodium glucose cotransporter 2 inhibitor; VA = ventricular arrhythmia.


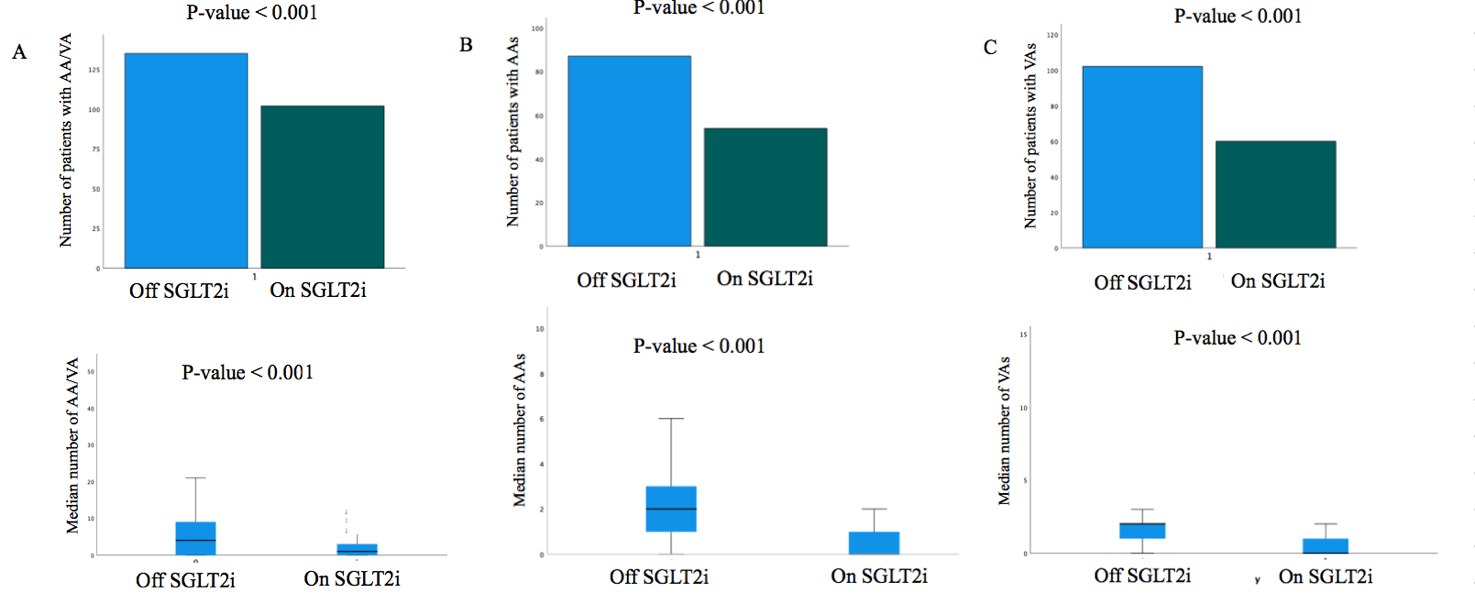

Supplement: Supplementary file 1 — Table S1. Number of arrhythmias pre‐ and post‐SGLT2i therapy in patients with baseline arrhythmic events. Table S2. Total, atrial and ventricular events in patients taking beta‐blockers, ACE‐I/ARB/ARNI and MRA during the study period. Table S3. Total, atrial and ventricular events in patients not taking beta‐blockers, ACE‐I/ARB/ARNI and MRA during the study period. Table S4. Total, atrial and ventricular events in patients taking amiodarone during the study period. Table S5. Total, atrial and ventricular events in patients taking sotalol during the study period. Table S6. Total, atrial and ventricular events in patients introducing amiodarone after SGLT2i prescription. Table S7. Total, atrial and ventricular events in patients introducing sotalol after SGLT2i prescription. Table S8. Total, atrial and ventricular events in patients off AADs during the study period. Table S9. Subgroup analysis: primary and secondary outcomes in patients without diabetes mellitus. Table S10. Subgroup analysis: primary and secondary outcomes in patients with diabetes mellitus. Table S11. Subgroup analysis: primary and secondary outcomes in patients with ischaemic cardiomyopathy. Table S12. Subgroup analysis: primary and secondary outcomes in patients with non‐ischaemic cardiomyopathy. Table S13. Subgroup analysis: primary and secondary outcomes in patients without CRT. Table S14. Subgroup analysis: primary and secondary outcomes in patients with CRT. [file EHF2-12-2125-s001.docx]
