# Supplementary figures and images for: SGLT2i reduce arrhythmic events in heart failure patients with cardiac implantable electronic devices
Source: ESC Heart Fail. 2025 Feb 7;12(3):2125–33. doi: 10.1002/ehf2.15223 (PMC12055389; doi:10.1002/ehf2.15223)

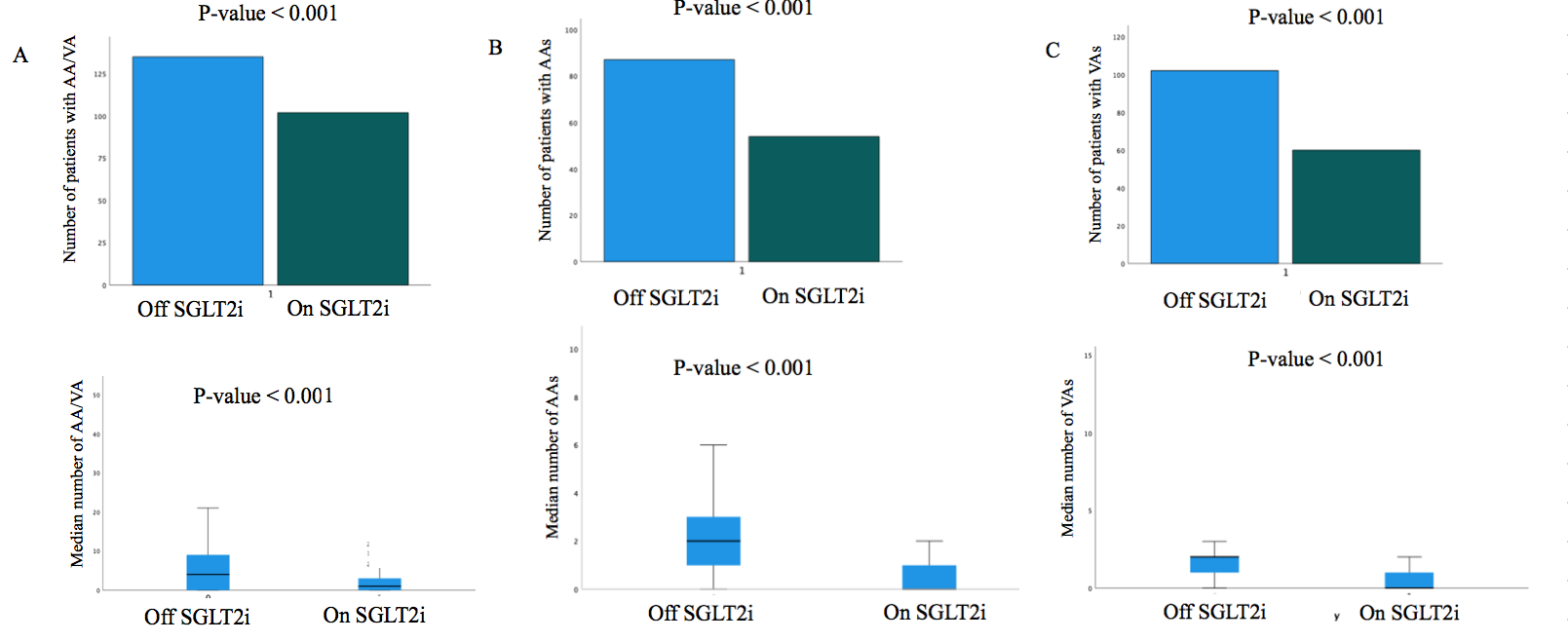

Supplement: Supplementary file 2 — Figure S1. Bar graphs and box plots showing the rate of arrhythmic episodes before and after SGLT2i therapy initiation. [file EHF2-12-2125-s002.tiff]
